# Mitral valve apparatus: echocardiographic features predicting the outcome of percutaneous mitral balloon valvotomy

**Published:** 2007-07

**Authors:** R Du Toit, EAW Brice, JD Van Niekerk, AF Doubell

**Affiliations:** Cardiology Unit, Department of Internal Medicine, Tygerberg Hospital, University of Stellenbosch; Cardiology Unit, Department of Internal Medicine, Tygerberg Hospital, University of Stellenbosch; Cardiology Unit, Department of Internal Medicine, Tygerberg Hospital, University of Stellenbosch; Cardiology Unit, Department of Internal Medicine, Tygerberg Hospital, University of Stellenbosch

## Abstract

**Objectives:**

To evaluate the significance of involvement of subvalvular apparatus in the outcome of percutaneous mitral balloon valvotomy (PMBV) in patients with mitral stenosis (MS) and to determine the predictive value of chordal length compared with current echocardiographic scores.

**Methods:**

Patients with significant MS were selected according to the Massachusetts General Hospital score (MGHS). Chordal lengths were assessed as additional markers of disease. Standard percutaneous valvotomies were performed. Valve area was assessed post-procedure with follow-up over one year.

**Results:**

Thirty-nine patients were prospectively studied. Valve area increased from a mean (SD) 0.97 (0.26) cm^2^ to 1.52 (0.38) cm^2^ with procedural success in 31 (79.5%) patients. There was no correlation (*r* = 0.09) between the MGHS and final valve area (FV A). There was a positive correlation between anterior chordal length and FV A (*r* = 0.66; *p* = 0.01). An FV A ≥ 1.5 cm^2^ was associated with higher mean chordal lengths (*p* = 0.01). A positive correlation was seen between valve area pre-procedure and FV A (*r* = 0.61; *p* < 0.01).

**Conclusions:**

The MGHS is valuable in the selection of patients for PMBV, but fails to separate selected patients into prognostic groups. Assessment of chordal length provides useful additional information, predicting the outcome of PMBV more accurately. Our data may support the earlier use of PMBV (asymptomatic patients).

## Summary

Mitral stenosis (MS) is a mechanical disorder, with a natural history altered only by surgery or percutaneous balloon valvotomy. 1,2 Medical management strategies are therefore viewed as adjunctive to further intervention when appropriate.

Mechanical intervention: The introduction of percutaneous transseptal mitral balloon valvotomy (PMBV) by Inoue and colleagues in 1984 gave a new option of treatment for patients with MS.^3^ In patients judged to have valve morphology suitable for a commissurotomy, several randomised trials have shown that balloon dilation gives results equivalent to open and closed surgical valvotomy, both short and long term.^4^-^7^ It has also proven to be a valuable therapeutic option in patients not fit to undergo mitral valve surgery.

Echocardiography is an essential non-invasive diagnostic procedure used to assess the severity of MS and to judge the feasibility of balloon valvotomy in a specific patient. To help predict which patients would have an optimal outcome after PMBV, several echocardiographic scoring systems have been developed.

The Massachusetts General Hospital score (MGHS) is a widely used echocardiographic scoring system developed by Wilkins *et al.*^8^,^9^ It assigns a severity grade from 0 to 4 to each of the following valvular morphological and functional characteristics: leaflet mobility, leaflet thickening, valvular calcification and subvalvular disease (ie, degree of chordal thickening). A high score (> 8 is associated with a lower immediate success rate and a higher rate of restenosis. Evaluation is done using precordial transthoracic echocardiography. This is also the scoring system currently in use at our cardiology department.

The ability of the MGHS to separate individual patients selected for PMBV into different prognostic groups appears to be limited.^9^ Echocardiographic underestimation of the severity of subvalvular involvement may contribute to the failure to identify a subgroup of patients who are at risk of a less-thanoptimal outcome and/or development of significant mitral regurgitation.^10^

The aim of our study was to identify those features of the mitral valve apparatus that would allow better selection of patients, increasing the proportion of patients with a successful outcome. We emphasised assessment of the subvalvular apparatus, focusing on chordal length as a predictor of outcome.

Transoesophageal echocardiography has been shown to provide a more accurate diagnostic evaluation of the mitral valve apparatus, an approach that is not currently used prior to PMBV for this specific purpose.^11^,^12^ We have therefore included combinations of different echocardiographic views in evaluating the subvalvular apparatus in our study.

## Patients and methods

A cohort study was done at the Cardiology Department of Tygerberg Hospital, University of Stellenbosch. A protocol was submitted to and approved by the ethics committee of the University of Stellenbosch. All patients consented to the study protocol, which included a description of the procedure. The study was conducted in accordance with the Declaration of Helsinki.

Between January 2002 and December 2003, 39 patients with symptomatic MS underwent PMBV. They had moderate/severe MS (valve area of ≤ 1.5 cm^2^ or mean transvalvular gradient of > 12 mmHg), a MGHS of < 10, no or mild MR and no contraindication to proceed with PMBV.

Prior to undergoing PMBV, all patients had repeat echocardiographic evaluation with two-dimensional and Doppler studies, using a Hewlett Packard Series 2000 ultrasound scanner. A 2.0/2.5-MHz probe and Hewlett Packard omni-plane probe were used for transthoracic, transoesophageal and transgastric views.

Standard two-dimensional transthoracic echocardiographic images were obtained in the parasternal long- and short-axis views and the apical four-chamber and long-axis views. Mitral valve area was calculated by direct two-dimensional planimetry. The resting mean pressure gradients across the mitral valve, as well as pulmonary artery pressure were recorded. Mitral valve morphology was scored as described by Wilkins and colleagues in the MGHS with a maximum score of 16.^8^ The left ventricular length (mm) was measured at the end of diastole using the apical four-chamber view.

The left atrium was evaluated using transoesophageal echo for the presence of thrombi, a possible contraindication for PMBV.13 The degree of commissural fusion was assessed as mild, moderate or severe.

**Fig. 1.**
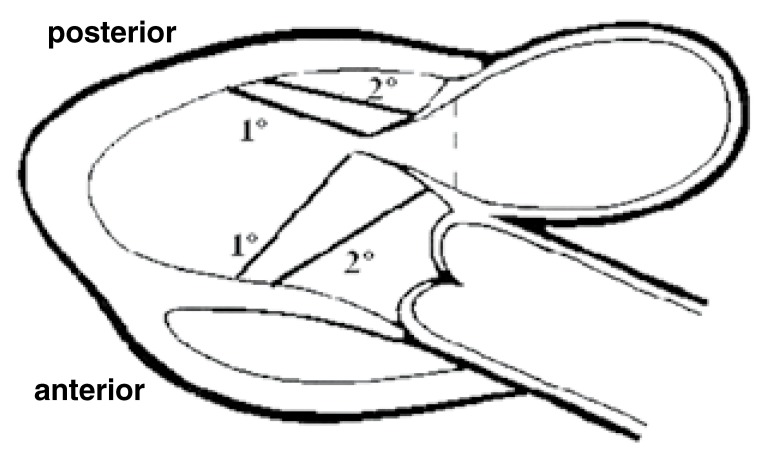
Transgastric echo (long-axis gastric view): length of primary and secondary chordae tendineae (mm).

The subvalvular apparatus was viewed using transgastric echocardiography in the long-axis gastric view. The length of the primary and secondary chordae tendineae was measured (mm) at the end of diastole (Fig. 1) and expressed as an absolute value as well as a ratio of left ventricular length.

Inoue percutaneous balloon mitral valvotomy was performed using the anterograde transseptal approach with a Brockenbrough needle (access via the femoral vein). The maximal nominal inflated balloon diameter was selected according to the patient’s height [height (cm) ÷ 10 + 10]. Balloon inflation was done in increments up to the maximal diameter. The end-point was an adequate transmitral gradient reduction or the development of significant mitral regurgitation.

## Assessment of results

Transthoracic echo and Doppler pressures were repeated post valvotomy. An immediate optimal result included: a final valve area of ≥ 1.5 cm^2^ or ≥ 50% increase in mitral valve area. Significant complications were regarded as severe mitral regurgitation, pulmonary:systemic shunt ≥ 2:1 across the iatrogenic atrial septal defect, systemic embolism, cardiac tamponade and death. Clinical and echocardiographic follow-up were done over a one-year period with assessments repeated on day one, and one month, six months and 12 months after the procedure. Endpoints were significant mitral valve restenosis (repeat valvotomy or mitral valve replacement) and cardiac death.

## Statistical analysis

Results were expressed as mean (SD). Spearman correlations were calculated to compare continuous variables. The Kruskal-Wallis test was used to determine significant differences between the means of different groups. Receiver operating characteristic (ROC) curves were used to determine the diagnostic ability of various measurements. A *p*-value < 0.05 was considered as statistically significant.

## Results

## Patient population and pre-procedural variables

The study population consisted of 39 patients, two male and 37 female, with a mean (SD) age of 36 (13) years (range 23−78). Ten patients (26%) were pregnant at the time of intervention and four patients (10%) were known to have had previous valvotomies. Four patients (10%) were in NYHA functional class I, 17 (44%) in NYHA class II, 10 (26%) in NYHA class III and six (15%) in NYHA class IV. Atrial fibrillation was present in six patients (15%). In one patient an emergency PMBV was done. Chordal lengths were therefore not recorded in this patient. Echocardiographic features prior to PMBV are summarised in Table 1.

**Table 1:** Echocardiographic Features Prior To PMBV

*Variable*	*MGHS*	*PAP (mmHg)*	*MVA (cm^2^)*	*Mean gradient (mmHg)*	*Anterior primary chordae (mm)*	*Anterior secondary chordae (mm)*	*Posterior primary chordae (mm)*	*Posterior secondary chordae (mm)*
*n*	39	39	39	39	38	17	38	23
Mean	6.620	46.41	0.97	13.38	19.67	18.08	15.22	12.24
Std deviation	1.41	17.43	0.26	5.52	7.29	8.25	6.24	3.66

MGHS, Massachusetts General Hospital Score; MVA, mitral valve area; PAP, pulmonary artery pressure; PMBV, percutaneous mitral balloon valvotomy.

## Clinical and echocardiographic follow-up

After a period of two years, one-month follow-up information was available for 34 (87%) patients, while 15 patients (38%) had reached their 12-month follow-up date; six patients (15%) were lost to follow-up at the time (Table 2). Over the follow-up period, varying from one to 12 months, there were no deaths; one patient was referred for mitral valve replacement due to the development of severe mitral regurgitation with symptomatic deterioration.

**Table 2:** Clinical And Echocardiographic Follow-Up

	*Pre-valvotomy*	*1 month*	*12 months*
Mean NYHA class (SD)	2.36 (1.02)	1.54 (0.89)	1.2 (0.94)
Mean MVA (SD) (cm^2^)	0.96 (0.26)	1.52 (0.28)	1.54 (0.39)
Mean transvalvular gradient (SD) (mmHg)	13.38 (5.52)	6.51 (2.23)	5.55 (2.63)
Mean PAP (SD) (mmHg)	46.41 (17.43)	37.06 (11.26)	36.8 (11.03)
Severe MR	0	2 patients (6%)	0

MR, mitral regurgitation; MVA, mitral valve area; NYHA, New York Heart Association; PAP, pulmonary artery pressure.

Immediate outcome: Inoue balloon mitral valvotomy was performed in all patients. The mean (SD) mitral valve area increased from 0.97 (0.26) cm^2^ to 1.52 (0.38) cm^2^ and the mean (SD) transvalvular gradient decreased from 13.38 (5.52) mmHg to 6.43 (3.21) mmHg. Procedural success (either a final valve area of ≥ 1.5 cm^2^ or a ≥ 50% increase in mitral valve area) was seen in 31 patients (79.5%) with a sub-optimal result in eight patients (20.5%). No immediate complications occurred. Mean (SD) pulmonary artery pressure was reduced from 46.41 (17.43) mmHg to 40 (13.43) mmHg.

## Predictors of outcome

There was a poor correlation (*r* = 0.09) between the MGHS and the final valve area achieved (Fig. 2a). When comparing the MGHS as a continuous variable with a final valve area of < 1.5 cm^2^ and ≥ 1.5 cm^2^, there was no significant difference in the mean MGHS of these two groups (Fig. 2b; *p* = 0.38).

**Fig. 2a.**
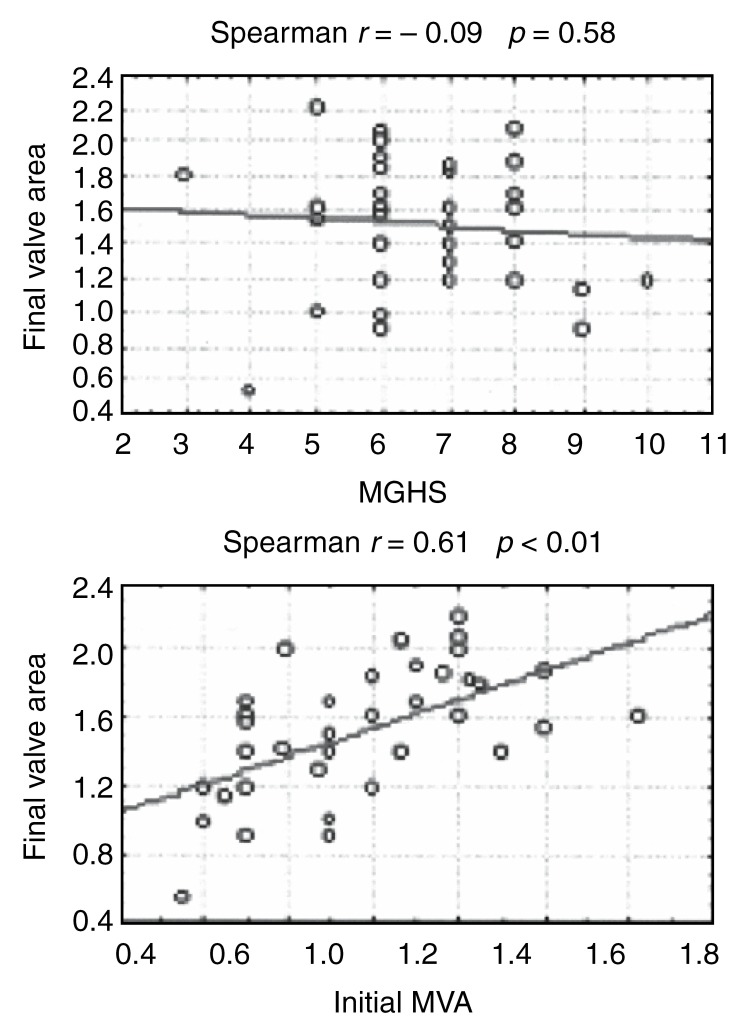
Scatter plot of final valve area (cm^2^) vs MGHS and initial MVA (cm^2^), respectively.

**Fig. 2b.**
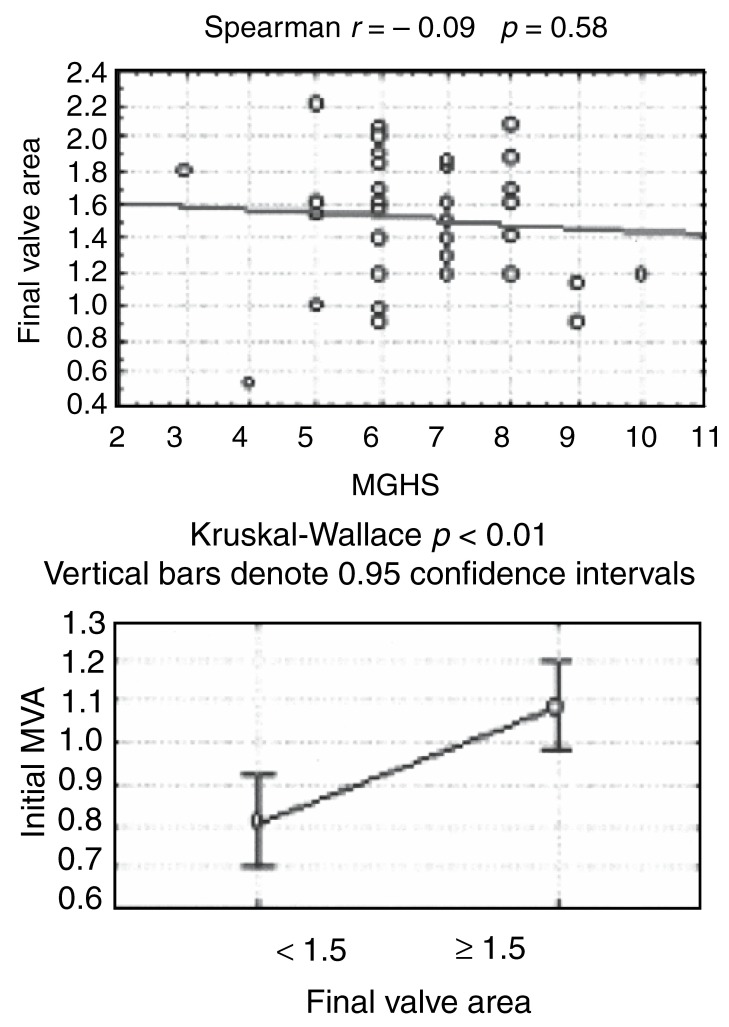
Average MGHS and initial MVA (cm^2^) in patients with a final valve area of < 1.5 and ≥ 1.5 cm^2^, respectively.

A positive correlation (*r* = 0.61; *p* < 0.01) was demonstrated between the initial mitral valve area and the final valve area achieved when compared as continuous variables (Fig. 2a). A final valve area of ≥ 1.5 cm^2^ was also associated with a significantly higher mean initial valve area (Fig. 2b; *p* < 0.01).

In evaluating the subvalvular apparatus, chordal lengths as continuous variables were correlated with the final valve area achieved. The four different chordae measured all showed a positive correlation with the final valve area (Fig. 3a). A final valve area of ≥ 1.5 cm^2^ was also associated with higher mean chordal lengths (Fig. 3b). Expressing the chordal length as a ratio of left ventricular length, to correct for patient size, did not have any significant influence on the above results.

**Fig. 3a.**
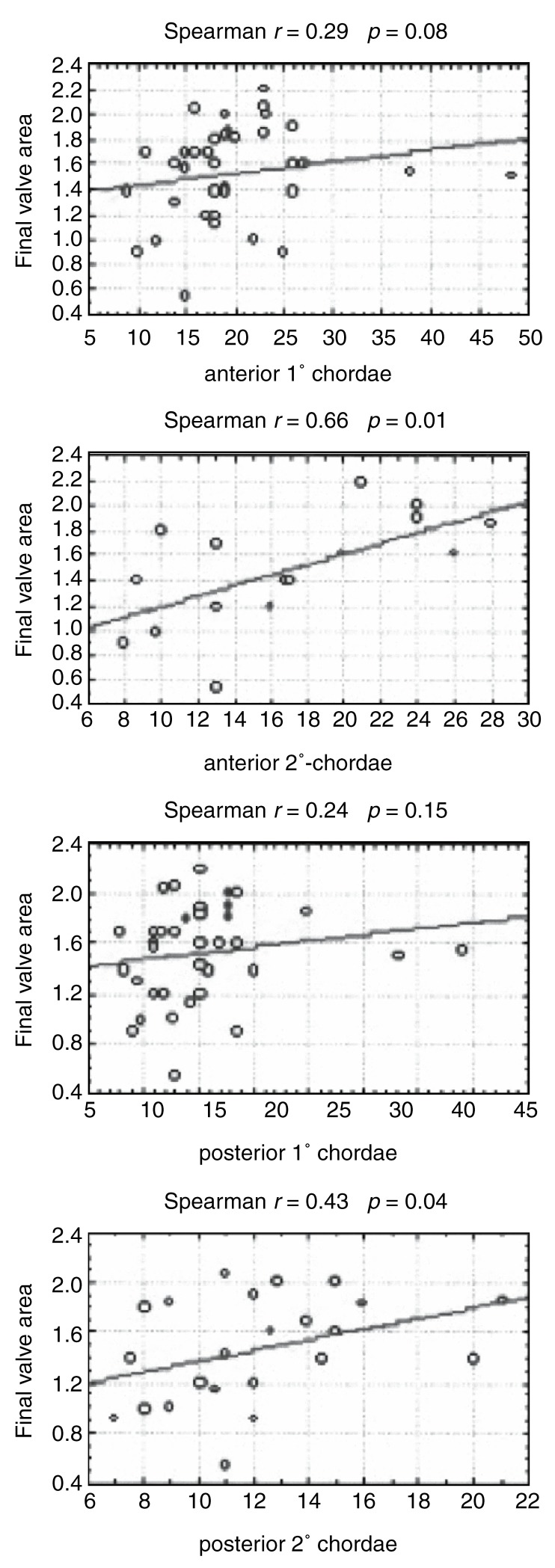
Scatter plot of final valve area (cm^2^) vs chordae tendineae (mm).

**Fig. 3b.**
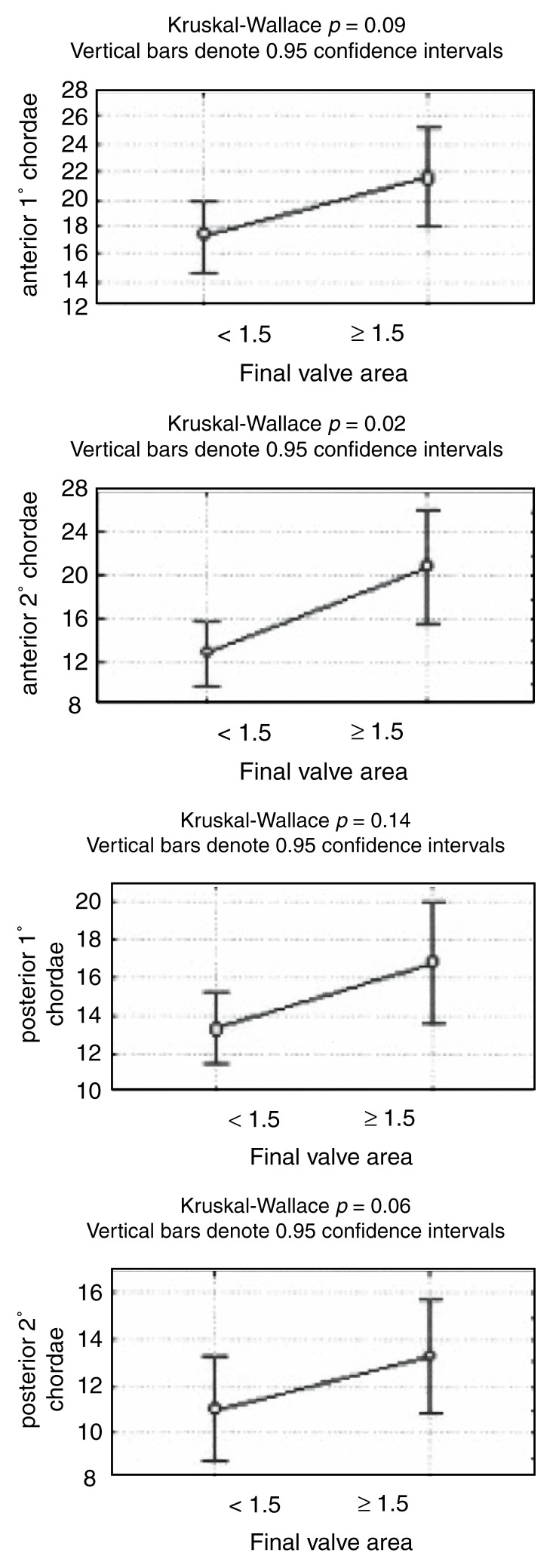
Average chordal lengths in patients with a final valve area of , 1.5 and ≥ 1.5 cm^2^, respectively.

**Fig. 4.**
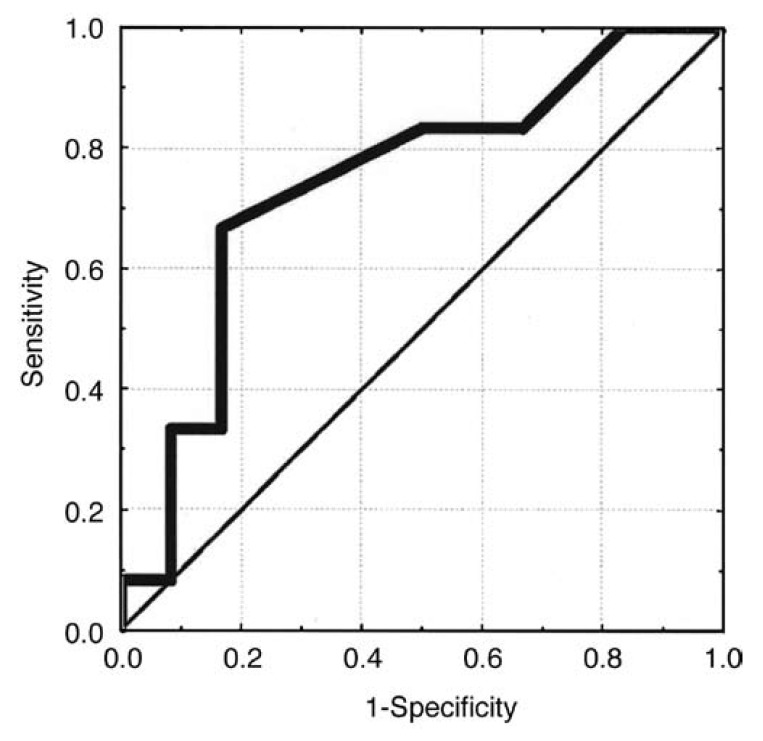
ROC curve using chordal length to predict a final valve area of ≥ 1.5 cm^2^. A posterior secondary chordal length of ≥ 12 mm has a sensitivity of 70% and specificity of 80% to predict a final valve area of ≥ 1.5 cm^2^.

In view of the positive correlations found between chordal length and the final valve area achieved, ROC curves were used to calculate the sensitivity and specificity of different chordal lengths to predict a final valve area of ≥ 1.5 cm^2^. A posterior secondary chordal length of ≥ 12 mm predicted a final valve area of ≥ 1.5 cm^2^ with a sensitivity of 70% and specificity of 80% (Fig. 4), while the other chordal measurements did not contribute to the predictions.

## Discussion

Mitral balloon valvotomy is a well-established nonsurgical alternative in the treatment of significant mitral stenosis. Although clinical variables are helpful in the initial identification of patients who require intervention, the strongest independent predictor of outcome in several studies has remained the morphological appearance of the mitral valve and the subvalvular apparatus.^8^,^9^

The use of an echocardiographic scoring system, the MGHS developed by Wilkins *et al*, has been accepted by most centres as an aid to patient selection, helping to predict both immediate and long-term outcomes.^8^,^9^ Our study confirms that the MGHS is valuable in the initial selection of patients who are likely to undergo an uncomplicated procedure with a successful outcome, seen in 31 (79.5%) of our 39 patients. The ability of the MGHS to predict a final valve area of ≥ 1.5 cm^2^ in individual cases was, however, limited (Fig. 2b) and the correlation with the final valve area achieved was poor (*r* = 0.09; Fig. 2a). This emphasises the limitations of the MGHS to separate selected patients into different prognostic groups.

Thickening and shortening of the subvalvular apparatus has been shown to be associated with a sub-optimal outcome, often due to the development of mitral regurgitation.^14^-^16^ Evaluation of the subvalvular apparatus using the MGHS focuses mainly on the degree of chordal thickening. The correlation between pathological specimens (*post mortem*) and the echocardiographic assessment is, however, poor.^17^ Underestimation of the severity of subvalvular involvement would contribute to the failure to identify a subgroup of patients who are at risk of a less-than-optimal outcome and/or development of significant mitral regurgitation.^9^,^10^

As a more objective evaluation of the subvalvular apparatus, we measured the length of each of the chordae tendineae and also expressed this as a ratio of the left ventricular length, to correct for patient size. The chordal lengths showed a correlation with the final valve area achieved, with the anterior secondary chordae showing the strongest (*r* = 0.66; *p* = 0.01) and the posterior primary chordae the weakest (*r* = 0.24; *p* = 0.15) correlation (Fig. 3a). A final valve area of ≥ 1.5 cm^2^ was associated with longer chordae tendineae: a final valve area of < 1.5 cm^2^ was associated with a mean anterior secondary chordal length of 12.79 mm versus 20.75 mm seen in patients with a final valve area of ≥ 1.5 cm^2^ (*p* = 0.02; Fig. 3b). Expressing the chordal length as a ratio of left ventricular length rather than absolute length did not make a significant difference to the above results.

Shortening of the chordae tendineae provides us with an objective echocardiographic measurement, reflecting the severity of subvalvular disease. Incorporation of chordal length in our future assessment of patients for PMBV by using an extended echocardiographic scoring system could improve the accuracy of selection of suitable patients.

Current indications for intervention (either PMBV or surgery) in patients with MS include moderate to severe MS (valve area < 1.5 cm^2^) in the presence of symptoms or, in the asymptomatic patient, significant pulmonary hypertension or pregnancy.^18^ The positive correlation demonstrated in our study between the initial mitral valve area and the final valve area achieved (Fig. 2a; *r* = 0.61; *p* < 0.01) as well as the significantly larger mean initial mitral valve area (Fig. 2b; *p* < 0.01) associated with a final valve area of ≥ 1.5 cm^2^ are most likely explained by basic mathematical principles (ie, the larger the starting value, the larger the final value). However, it does raise the question whether PMBV shouldn’t be considered in the asymptomatic patient with moderate MS (mitral valve area of 1−1.5 cm^2^), even in the absence of significant pulmonary hypertension or pregnancy. Whether this initial successful outcome will translate into a sustained favourable clinical and haemodynamic outcome will require further evaluation in a prospective study.

A relative small number of patients (39) were studied and the lack of complete data for all patients was a limitation. Visualisation of chordal structures requires optimal echocardiographic windows. Combinations of transgastric and transthoracic views were required in some patients to evaluate the chordae accurately. In patients with severe damage to the subvalvular apparatus, the secondary chordae tendineae were not always identified, limiting the number of patients in the analysis.

## Conclusions

Echocardiographic morphology, as described by Wilkins *et al.* in the MGHS, remains a valuable guide in our selection of patients suitable for PMBV. A final decision to perform the procedure in an individual patient should, however, include other considerations.

The length of the chordae tendineae provides an objective reflection of subvalvular disease, correlating with immediate outcome after PMBV. Although this feature has not been evaluated as a lone predictor of outcome in the unselected patient, its addition to current scoring systems may allow better selection of patients (ie, identify patients at risk to have a sub-optimal result), aiding in the decision of medical versus surgical/interventional therapy in a selected individual. Future application of this echocardiographic information through the development of a new or additional scoring system could increase the proportion of selected patients with a successful outcome after PMBV.

In view of the clear correlation found between the initial valve area and the final valve area achieved, future consideration through prospective studies should be given to the earlier use of PMBV in asymptomatic patients with moderate MS before progression to more severe disease.
